# *Dictyocoela* microsporidia diversity and co-diversification with their host, a gammarid species complex (Crustacea, Amphipoda) with an old history of divergence and high endemic diversity

**DOI:** 10.1186/s12862-020-01719-z

**Published:** 2020-11-11

**Authors:** Adrien Quiles, Rémi A. Wattier, Karolina Bacela-Spychalska, Michal Grabowski, Thierry Rigaud

**Affiliations:** 1 Université Bourgogne Franche-Comté, Laboratoire Biogéosciences, UMR CNRS 6282, 6 boulevard Gabriel, 21000 Dijon, France; 2 Department of Invertebrate Zoology and Hydrobiology, Faculty of Biology and Environmental Protection, University of Łódź, Banacha 12/16, 90-237, Łódź, Poland

**Keywords:** Host-parasite relationships, Co-diversification, Phylogeny, SSU rDNA, *Gammarus balcanicus*, Microsporidia

## Abstract

**Background:**

Although the processes of co-evolution between parasites and their hosts are well known, evidence of co-speciation remains scarce. Microsporidian intracellular parasites, due to intimate relationships with their hosts and mixed mode of transmission (horizontal but also vertical, from mother to offspring), may represent an interesting biological model for investigating co-speciation. Amphipod crustaceans, especially gammarids, are regular hosts of microsporidian parasites, in particular the *Dictyocoela* spp., which have so far been found limited to these amphipods and are known to use a vertical mode of transmission. The amphipod genus *Gammarus* has a diversification history spanning the last 50–60 Mya and an extensive cryptic diversity in most of the nominal species. Here, we investigated the degree of co-diversification between *Dictyocoela* and *Gammarus balcanicus*, an amphipod with high degrees of ancient cryptic diversification and lineage endemism, by examining the genetic diversity of these parasites over the entire geographic range of the host. We hypothesised that the strong host diversification and vertical transmission of *Dictyocoela* would promote co-diversification.

**Results:**

Using the parasite SSU rDNA as a molecular marker, analyzing 2225 host specimens from 88 sites covering whole host range, we found 31 haplogroups of *Dictyocoela*, 30 of which were novel, belonging to four *Dictyocoela* species already known to infect other *Gammarus* spp. The relationships between *Dictyocoela* and gammarids is therefore ancient, with the speciation in parasites preceding those of the hosts. Each novel haplogroup was nevertheless specific to *G. balcanicus*, leaving the possibility for subsequent co-diversification process during host diversification. A Procrustean Approach to Co-phylogeny (PACo) analysis revealed that diversification of *Dictyocoela* was not random with respect to that of the host. We found high degrees of congruence between the diversification of *G. balcanicus* and that of *Dictyocoela roeselum* and *D. muelleri*. However, we also found some incongruences between host and *Dictyocoela* phylogenies, e.g. in *D. duebenum*, probably due to host shifts between different *G. balcanicus* cryptic lineages.

**Conclusion:**

The evolutionary history of *Dictyocoela* and *Gammarus balcanicus* represents an example of an overall host-parasite co-diversification, including cases of host shifts.

## Background

The intimate relationships between symbionts and their hosts sometimes suggest that any event of speciation in the host lineage is likely to result in the simultaneous isolation event of species in its associated symbionts [1–3]. Such co-speciation patterns have been found mostly between mutualistic symbionts and their hosts [3], a phenomenon facilitated by some peculiar ecological conditions or life-history traits, such as by symbiont vertical transmission [4], but see [5]. Cases of co-speciation between parasites and their hosts seems rarer [3]. However, despite probable overestimates of this phenomenon [3], recent studies suggest its occurrence in various parasites taxa [2, 6, 7].

Because of their intimate relationships with their hosts, microsporidian parasites have been suggested as good candidates for showing some degrees of co-speciation, for example in *Culex* mosquitoes infected by *Amblyospora* microsporidia [8]. Microsporidia are obligate unicellular endoparasites belonging to an extremely ancient and phylogenetically diverse phylum close to fungi [9]. These ubiquitous parasites infect a wide range of vertebrate and invertebrate hosts [10]. They are responsible for many diseases of insects and crustaceans [11–13]. Among aquatic arthropods, the freshwater amphipod crustaceans, especially those of the superfamily Gammaroidea, are commonly infected by microsporidia (for overviews see [12, 14, 15]). *Nosema* [16], *Cucumispora* [17] and *Dictyocoela* [18] commonly infect numerous gammarid species across Eurasia [18–21] and North America [22, 23].

In particular, *Dictyocoela* spp. form a monophyletic clade restricted to amphipod hosts. Many *Dictyocoela* species, or strains within species has been identified, mostly using molecular tools [18–21, 24–28]. However, a number of these variants were formally described as species using both molecular and morphological criteria [23, 24], namely *Dictyocoela muelleri*, *D. roeselum*, *D. berillonum*, *D. duebenum* and *D. diporeiae*. Most of them include some degrees of intra-taxa molecular divergence. These microsporidia infect a wide range of amphipod species, specifically *Gammarus* spp. [24, 29]. The life cycle of numerous *Dictyocoela* species is not known. In two hosts, *Gammarus duebeni* and *G. roeselii*, *Dictyocoela roeselum*, *D. duebenum* and *D. muelleri* are known to be vertically transmitted: they infect oocytes, and, therefore, are transmitted to most embryos [30–32]*.* These species induce low virulence [33] as well as sex-ratio distortion in their host populations. Indeed, the putative male host receiving the vertically transmitted parasites are reversed into functional females [34–36]. However, some strains of *Dictyocoela duebenum* are not sex ratio distorters [35], and almost all most of the strains of the species described by Bacela-Spychalska et al. [24] were also found to infect muscles. It indicates possible variation in life-history traits, such as transmission pathway or feminization, among *Dictyocoela* and/or according to the host species.

Most of the phylogenetic studies suggest little co-speciation between gammarids and the *Dictyocoela* species identified so far. Studies by [37] and [24] both showed that all these species of infect many host taxa. For example, *Dictyocoela duebenum*, discovered in the Northwestern European *Gammarus duebeni*, has been found infecting the Ponto-Caspian *Dikerogammarus villosus*, the Southwestern European *Echinogammarus berilloni* as well as the Baikal hosts *Gmelinoides fasciatus* and *Brandtia latissima*. Similarly, *Dictyocoela muelleri* infects the Ponto-Caspian gammarids *D. villosus* and *Pontogammarus robustoides*, plus *Gammarus duebeni*, *G. roeselii* and *G. varsoviensis*, the latter three species being from Northwestern, Southeastern and Central Europe, respectively [24]. However, within each of these parasite species, a single *Dictyocoela* strain (based on the partial or total sequences of the SSU rDNA sequence) rarely share two host species. It, therefore, remains challenging to understand whether some *Dictyocoela* variants are specific for some hosts, or if *Dictyocoela* spp. are generalist parasites. The difficulty mainly comes from the fact that many of these studies were not based on the extensive sampling of hosts, but were based on rather punctual samples over large geographic areas or samples from limited geographic areas. For example, [20] showed that several species of hosts are infected by several microsporidian species/clades at a small geographic scale (a river drainage in the Ruhr region in Germany). Similarly, [26] showed that, in the peculiar context of the Lake Baikal gammarid radiation, microsporidia did not follow the differentiation of their hosts, but can instead be seen as generalist parasites exploiting different hosts. Moreover, host-parasite exchanges between Lake Baikal and surrounding rivers are frequent in this ecosystem. Conversely, at a larger scale, an extensive study of a single host (*G. roeselii*) over its whole geographic range suggests that there may be some degree of specificity within *Dictyocoela roeselum*, with some strains of this parasite species being restricted to infect *G. roeselii* only [27]. However, other microsporidia species infecting this host result probably from host-shifts, from local amphipod fauna, following *G. roeselii* colonization of new geographic areas. More data of such kind are therefore needed to draw a more general picture of *Dictyocoela*-gammarid associations. A recent analysis of microsporidia in endemic New-Zealand amphipods indeed revealed an overall congruence between phylogenies of *Paracalliope* spp. and their *Dictyocoela* parasites. At a global scale, the observed pattern is similar between amphipods (beyond, but including, gammarids) and *Dictyocoela*. This pattern may have resulted from covicariance and/or codispersal, suggesting that the intimate association between amphipods and *Dictyocoela* may have persisted over macroevolutionary time [38].

**Fig. 1:**
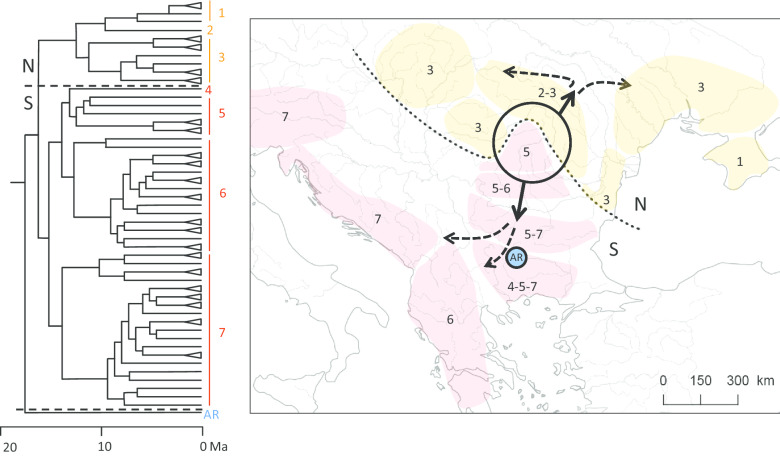
*Gammarus balcanicus* phylogeographic history (after [39]). Left: Maximum clade credibility chronogram generated using Bayesian inference and based on the multimarker data from Fig. S4 of [39]. Three major clades: *AR* Ancient Rhodope, *N* North-Eastern, *S* South-Western are identified. N and S were further subdivided into three (1–3) and six (4–7) clades, respectively. Right: Present day distribution of clades and biogeographic scenario of diversification presented by circles and arrows. Map created by authors using Qgis 2.18.4 (QGIS Development Team 2009). See text for details

Here, we add a third extensive census of *Dictyocoela* parasites in another gammarid host over its entire geographic range: *Gammarus balcanicus*, a morphospecies scarcely known for the presence of microsporidia. This host was chosen as a biological model for its high degree of diversification and divergence [39], making it particularly interesting to investigate parasite-host co-diversification. *Gammarus balcanicus* inhabits mountainous areas from the eastern Carpathians through the Balkan Peninsula, to the eastern Alps. However, some populations are also known from the Black Sea lowlands and in Crimea [39, 40]. The Carpathians and the Balkans are recognized as the most valuable present-day hot-spots of biodiversity and endemism, and a model system for studies upon biogeography and the evolution of numerous organisms [41, 42]. Notably, it is an ancient centre of diversity for some freshwater gammarids [39, 40, 43]. *Gammarus balcanicus* is characterized by high cryptic diversity, including at least 50 divergent MOTUs (Molecular Operational Taxonomic Units) of Miocene origin, clustered in seven main phylogenetic lineages (Fig. 1) [39]. Within these lineages, the present-day *G. balcanicus* MOTUs are locally endemic, due to their complex phylogeographical history and habitat fragmentation. In a caricatural way, we could say that each small river basin harbours a separate MOTU of *G. balcanicus*. Indeed, the Balkan Peninsula and the Carpathian Arch have been characterized by landscape remodeling and high dynamic geographical complexity [44]. *Gammarus balcanicus* species complex has started its diversification at *ca.* 20 Ma, in the early Miocene in the central Balkans, partially in the shallow epicontinental sea of Paratethys [39]. This early diversification generated two major clades (Fig. 1): the AR clade, nowadays endemic to a small area in the Rhodope Mountains in the central Balkan Peninsula, and a huge clade which later (*c.* 15 Ma) split into the north-eastern clade (hereafter N) and the south-western clade (hereafter S). Subsequent diversification and geographic expansion of the north-eastern and south-western clades of *G. balcanicus* continued following the Alpine orogeny during Miocene/Pliocene and, finally, during the Pleistocene glaciations (Fig. 1) [39]. Such a history makes this species complex a perfect model to test host-parasite co-diversification events.

The high degree of diversification and endemicity within *G. balcanicus* complex may provide ideal conditions for parasite co-diversification. This supposition is strengthened by the fact that *Dictyocoela* spp. found so far in this gammarid are the vertically-transmitted *Dictyocoela muelleri* [18, 29] and *Dictyocoela roeselum* [24], observed sporadically in Carpathian populations. The vertical-transmission of symbionts makes host-parasite co-diversification more probable [3]. However, studies on microsporidian infections in *G. balcanicus* are too scarce to affirm that these parasites are the only ones infecting this species. Therefore, using PCR assays and DNA barcoding approach with the parasite SSU rDNA molecular marker, we investigated *Dictyocoela* spp. associated with *G. balcanicus*, in comparison with published data in other gammarids, to address the following issues: (1) Are there any *Dictyocoela* species or strains specific *to G. balcanicus* host? (2) Has the host phylogeographic history influenced host-parasite association and at what scale the co-diversifications can be observed between *G. balcanicus* and *Dictyocoela* parasites? Both the high degree of diversification and endemicity of the host and the vertical transmission of *Dictyocoela* should promote co-differentiation. This could occur at the scale of either between parasite species, if some *Dictyocoela* species infected *G. balcanicus* after its diversification, or between parasite strains within species, if associations between *G. balcanicus* and *Dictyocoela* is older than the host diversification. Owing to the results obtained on other gammarids at different geographic scales (see, e.g. [20, 24, 26]), we would predict the later hypothesis being more probable.

## Methods

### Sampling and total DNA extraction

**Fig. 2:**
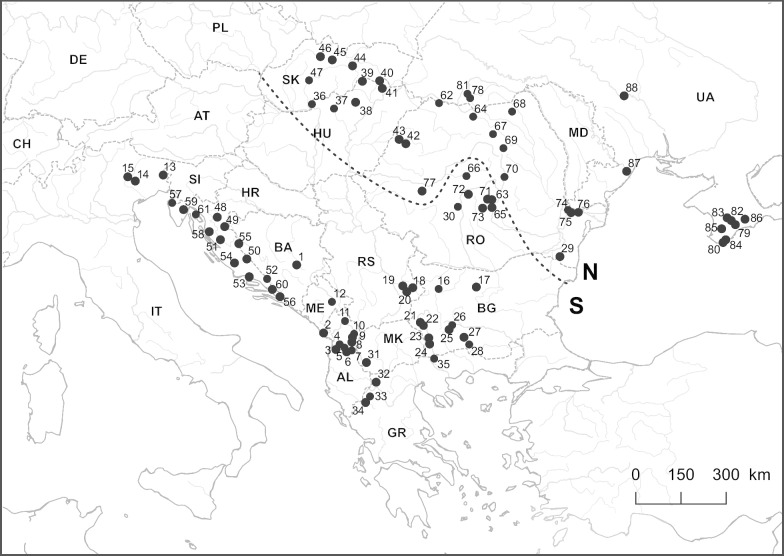
*Gammarus balcanicus* sampling sites. Sites are identified by numbers (1–88). See Additional file 1 for details (e.g. sampling sizes, GPS coordinates). The dashed line delimits the geographic distribution of the so-called N and S clades (see Fig. 1. and text for details). Map created by authors using Qgis 2.18.4 (QGIS Development Team 2009)

*Gammarus balcanicus* individuals were gathered during several sampling campaigns between 2004 and 2016, at 88 sites in 13 countries, covering the entire distribution range of this morphospecies in Europe (Fig. 2, Additional file 1). The sites were plotted on a map using Qgis 2.18.4 (QGIS Development Team 2009). Samples were collected using hand nets and kick-sampling method. All individuals were immediately fixed in 96% ethanol at the sampling site and stored at room temperature after returning to the laboratory. Amphipods were identified to the morphospecies level using morphological characters described in available keys (e.g. [45, 46]). Samples used in the present study correspond to the *G. balcanicus* species complex samples used by [39]. All the specimens are stored at the Department of Invertebrate Zoology and Hydrobiology, University of Lodz, Poland.

Each gammarid was dissected under a stereomicroscope. Approximately 2 mm^3^ of host tissue (including muscles and gonads) were taken from the 6th and 7th thoracic segments. Since microsporidia are intracellular parasites, their DNA was co-extracted with host DNA. Among the 2255 host individuals used in the present study, DNA was obtained from 1202 males and 1053 females (females were not available in all gathered samples). The DNA extraction was performed using either (i) standard phenol–chloroform protocol [47] or (ii) Biobasic EZ-10 96 Well Plate Genomic DNA Isolation Kit for Animal Sample and eluted in 100 µl of TE (pH 8). The DNA samples were kept at 4 °C until amplification and subsequently at − 20 °C for long-term storage.

### Molecular screening for microsporidia

All the 2255 individuals were screened for the presence of microsporidia following the strategy described in [27], using the short (c. 350 bp long) diagnostic fragment of the small ribosomal subunit gene (SSU rDNA), amplified with the microsporidia-specific primers V1f (forward) (5′-CAC CAG GTT GAT TCT GCC TGA C-3′) paired with UNIr (reverse) (5′-TCA GGC TCC CTC TCC GGA AT-3′) [27]. The use of this short fragment maximized the ability to detect the presence of microsporidians even in case of low infection intensity or partial degradation of DNA. As negative and positive controls in PCR reactions, we used, respectively, water and *Dictyocoela roeselum* DNA from *Gammarus roeselii* [27]. The PCR conditions and visualization of PCR products were as described by [27].

In individuals positively diagnosed for microsporidian infections our objective, following [27], was to sequence the ca. 800 bp long fragment of the SSU rDNA gene matching the 5′ part either as one or two overlapping fragments. When the 800 bp long sequence was not obtainable, we used either a V1f-530r fragment (c. 530 bp long) or even a V1f-UNIr fragment (c. 350 bp long), that contained enough phylogenetic information to attribute sequences to the species level without any ambiguity (Additional file 2). PCR products were purified and sequenced directly with the BigDye technology by Genewiz, Inc., UK, using the forward primers from PCR. Using Geneious 10.2. [48]. Raw sequences were edited and trimmed. Their microsporidian identity was confirmed using BlastN searches against sequences available in GenBank [49].

### Phylogeny reconstruction for microsporidians and taxonomic assignment of newly produced sequences

For SSU rDNA, our dataset is composed of two types of microsporidian sequences (Additional file 2). First, newly produced sequences from the infected *G. balcanicus* individuals. Second, literature SSU sequences of *Dictyocoela* spp. found infecting European freshwater or brackish water amphipods, as in [27], with the addition of parasites from Lake Baikal [19, 26, 28], and from USA [23, 50, 51]. Sequences were aligned using MAFFT7.388 software [52], with the E-IONS-I algorithm using the legacy gap penalty option, incorporated in Geneious 10.2.2 [48]. Our dataset contains sequences of different lengths both among the newly produced sequences and the published ones (Additional file 2). As some sequences were relatively short, reducing the full dataset to a standard size would, on the one hand, allow defining haplotypes but, on the other hand, would potentially induce losing the phylogenetic signal. Therefore, following the strategy described by [27], we attributed each sequence to a so-called haplogroup. Sequences belonging to distinct haplogroups harbored at least one or more variable sites, generating diagnostic features on the minimal length of a given haplogroup. Few sequences could not be assigned to only one haplogroup due to their short length and the resulting absence of diagnostic features. The longest sequence of each haplogroup was then used for the phylogeny reconstruction (Additional file 2). The best-fitting model of nucleotide substitution was determined with JModelTest-2.1.10 [53] as being the General Time Reversible (GTR) model with gamma-distributed rate heterogeneity (G) and a significant proportion of invariable sites (I). Phylogenetic reconstructions were built using the Maximum Likelihood algorithm implemented in Mega-X [54] using extensive subtree-pruning-regrafting as ML heuristic method with very strong branch swap filtering and 1000 boostraps. Four sequences, AF044391 (*Glugea anomala*), GQ203287 (*Glugea hertwigi*), GQ246188 (*Cucumispora dikerogammari*) and KR190602 (*Cucumispora ornata*) were used as outgroup. Such a tree was aiming two purposes. First it presented an overall view of the diversity and divergence observed in the genus *Dictyocoela*. However, this tree did not aiming to provide a supported topology of clades at the genus level, given the very large taxa sampling, the use of a single marker and sequence being variable in length. Second, this tree allowed the taxonomical assignement of newly detected *Dictyocoela* sequences based on their phylogenetic proximity to published sequences, especially to sequences associated with formerly described taxa, i.e. *D. muelleri, D. roeselum, D. berillonum* and *D. duebenum* [24]. In addition, a ML tree was built to provide support of the key clades presented in this paper. It was based on a subsample of individuals representative of diversity and divergence of all the *Dictyocoela* clades highlighted in the present paper, taking advantage of long SSU rDNA sequences, as well as ITS and LSU sequences when available. The tree was also based on GTR+G+I model, using 1000 bootstraps and the same outgroup. The topology of this tree is similar to the ones in [24] and [28].

### Congruence between parasite and host phylogenies

The overall link between divergence patterns of *Dictyocoela* spp. and *G. balcanicus* was tested using Procrustean Approach to Co-phylogeny (PACo), one of the most conservative methods for analyzing co-phylogenies [55]. PACo does not require fully resolved phylogenetic trees, which was the case for some parts of our parasite tree. This analysis can be based on genetic distances only. Therefore, we first constructed matrices of genetic distances for both the host and the parasites. For parasites, we used our newly produced sequences. For hosts, we used the COI (Cytochrome Oxidase I gene) haplotypes published by [39]. As often as possible, we took the haplotype of the individual we found infected (Additional file 1). However, since we did not know the haplotypes of all our infected individuals, we took the host haplotypes representative of the BIN (Barcode Index Number) present in the population. The BIN is implemented as part of the Barcode of Life Data system (BOLD; [56]. Sequences submitted to BOLD are clustered according to their molecular divergence and each cluster is ascribed a unique identifier (BIN), registered in BOLD. In our data set, there was one BIN per site, except for the HR05 site, where two BINs were present (Additional file 1). Since each BIN is a cluster of closely-related sequences, we took only one of these sequences as a representative of the BIN. For site # 50 (HR05), the two sequences were called 50 a and b. The matrices of genetic distances were computed with MEGA-X, using the Tamura-Nei substitution model (TN93, estimated by MEGA-X as being the best evolutionary model). These two distance matrices were linked by a binary matrix coding the host-parasite associations (see Fig. 5). Some parasites could be attributed to two haplogroups because of their short sequence length, resulting in losing some diagnostic sites (see above). We therefore constructed one binary matrix for each hypothesis. The ambiguous parasites were the following: For *D. muelleri*, matrices were build considering that individuals of the site # 56 (HR20) harbored a *D. muelleri* b07 haplogroup, or a b08 haplogroup. Similarly, gammarids from two sites (# 64, RO04 and # 74, RO36) harbored parasites of either *D. roeselum* b15 or b16 haplogroups. Different matrices were constructed to take this uncertainty into account (b15 in 64 – b16 in 74; b16- in 64 – b15 in 74; b15 in both sites; b16 in both sites). For each calculation, a residual sum of squares was then calculated as a global goodness-of-fit statistic between the host and parasite phylogenies (see [55] for details). Its significance was established by assigning hosts randomly to parasites on the parasite-host matrix with 1,000,000 permutations, testing the null hypothesis that there was no congruence between host and parasite phylogenies. The contribution of each host-parasite link to the global fit was assessed with a jackknife procedure that estimates the squared residual and its 95% confidence interval. The lower the residual, the higher its contribution to the global fit. For the sake of brevity and conservatism, in the main text and figures, we only presented the calculations for the hypothesis where the P values were the highest, all results being significant. However, the detailed results are provided in Additional file 3. The matrices used are also given in Additional file 3.

## Results

### *Dictyocoela* species found in *G. balcanicus* and comparison with those of other hosts

**Fig. 3:**
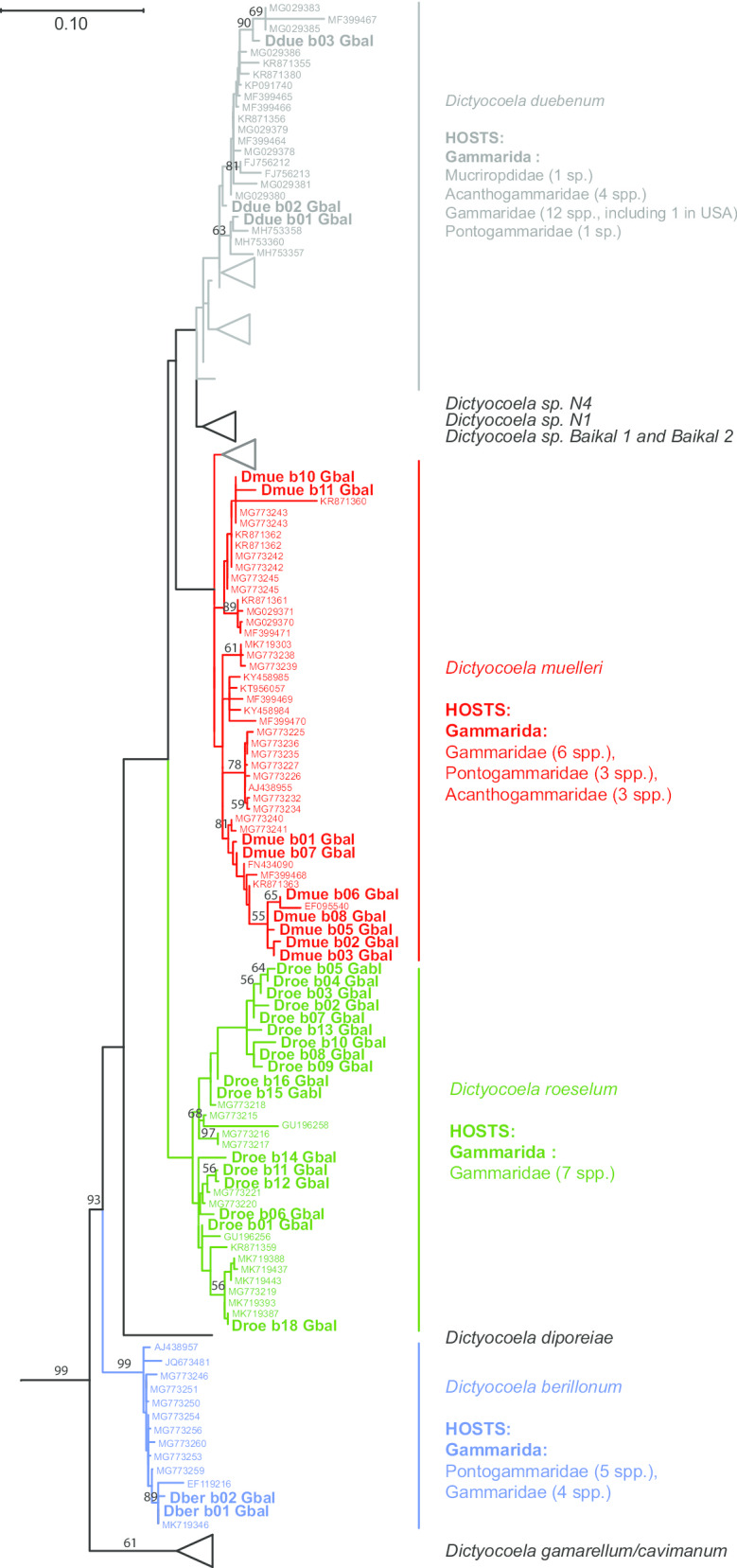
Maximum-likelihood phylogenetic reconstruction based on small ribosomal subunit (SSU) rDNA for the microsporidian genus *Dictyocoela*. The four taxa identified to infect *G. balcanicus* were shown in colors: *D. duebenum* (grey), *D. muelleri* (red), *D. roeselum* (green) and *D. berillonum* (blue). *Dictyocoela* are referred as their Genbank number (see Additional file 2), except haplogroups from the present study (in bold). Some sequences were collapsed as triangle, the size of which do not reflect within diversity and divergence (detailed phylogeny provided in Additional file 4). Four sequences, AF044391 (*Glugea anomala*), GQ203287 (*Glugea hertwigi*), GQ246188 (*Cucumispora dikerogammari*) and KR190602 (*Cucumispora ornata*) were used as outgroup (not shown on the tree, see Additional file 4). Values at nodes are bootstrap values > 50%

In the 2255 analyzed individuals of *G. balcanicus*, overall, 31 haplogroups of *Dictyocoela* spp. were found infecting 139 individuals in 45 sites (Additional file 1). When placed onto the global phylogeny of *Dictyocoela*, the haplogroups infecting *G. balcanicus* could be ascribed to four fully described species of *Dictyocoela* parasites according to [24]: *Dictyocoela roeselum*, *D. muelleri*, *D. duebenum* and *D. berillonum* (Additional files 4 and 5, Fig. 3). It is to be noticed that only one of these 31 haplogroups was shared with another gammarid host species (Droeb18, see later).

This global phylogeny confirms that few examples of specialization can be found in *Dictyocoela* spp: different host species or group of species shares most of these parasite species (Additional file 4). For example, the clade including *Dictyocoela cavimanum* and *D. deshayesum* is the only *Dictyocoela* clade infecting amphipods outside the infra-order Gammarida (sensu [57]) or gammaroids (sensu [58]) (Additional file 4). *Dictyocoela* of this clade infect Talitrida, an infra-order that diverged from gammaroids during early Mesozoic, ca. 180–200 MYA [58], and now distributed globally (e.g. *Hyallela* in America, *Orchestia* in Eurasia). However, parasites from this clade also infect a host species belonging to Baikal Acanthogammaridae (*Ommatogammarus flavus*) (Additional file 4). All other *Dictyocoela* infect only amphipods belonging to the Gammarida infra-order, but a single parasite species often infect host species from several gammarid subclades (as defined by [59]). For example, *D. muelleri* is found infecting species belonging to six clades: saline *Gammarus* group (*Gammarus duebeni, G. aequicauda*), *G. roeselii* group*, G. lacustris* group *(G. varsoviensis), G. balcanicus* group*, G. pulex* group, Ponto-Caspian group (sensu [60]) (*Dikerogammarus villosus, D. haemobaphes, Pontogammarus robustoides*) and three Baikal Acanthogammaridae (*Eulimnogammarus vittatus*, *E. verrucosus* and *Acanthogammarus lappaceus*) (Additional file 4). The host range of *D. duebenum* is even wider, including virtually all the host species groups of the Gammarida infra-order, but the Dinaric and Mediterranean *Echinogammarus* groups (Additional file 4). Despite its limited genetic diversity relative to other *Dictyocoala*, *D. berillonum* also has a wide host spectrum, with five host species groups infected (*G. balcanicus*, *G. roeselii*, pontogammarids, Marine and Atlantic *Echinogammarus* groups). Contrastingly, the host range of *D. roeselum* includes only *Gammarus* species from six different groups (*G. lacustris*, *G. roeselii*, *G. balcanicus*, *G. pulex*, *G. fossarum* and one saline *Gammarus*). Some of the *Dictyocoela* clades infecting Baikal amphipods seems restricted to this geographic area. Indeed, one *Dictyocoela* clade (*Dictyocoela* sp. Baikal 2) is limited to only one species of the oriental *Gammarus* group (*Gmelinoides fasciatus*) (Additional file 4). However, within this zone, some *Dictyocoela* species are also found in other geographic regions (*D. muelleri* and *D. duebenum*) (Additional file 4).

### Diversity of *Dictyocoela* infecting the *G. balcanicus* morphospecies

**Fig. 4:**
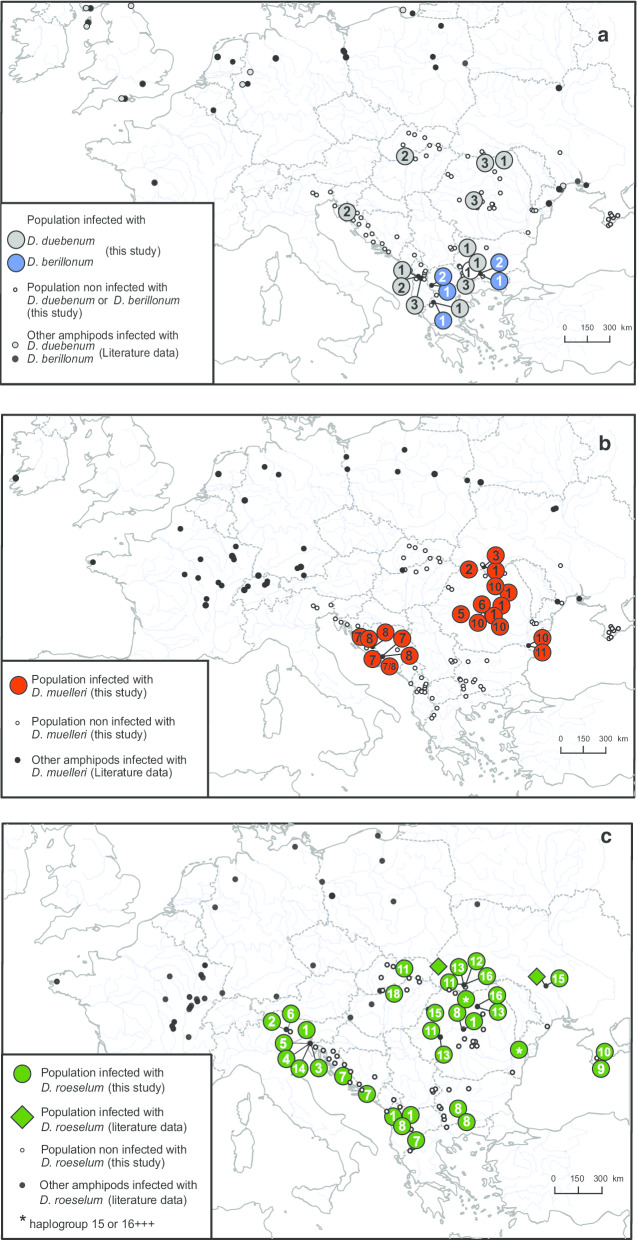
Geographic distribution of *Dictyocoela* spp infecting *Gammarus balcanicus.* Maps **a**, **b** and **c** refers to *Dictyocoela berillonum* (blue) and *D. duebenum* (grey), *D. muelleri* (red) and *D. roeselum* (green)*,* respectively. Each map shows: (i) sites with infections found in *G. balcanicus* (large colored dots including haplogroup numbers and diamonds, for present study and literature data, respectively), (ii) presence of parasites in other amphipods (for information, small black dots) and (iii) non-infected sites from this study (small empty dots) (see Additional files 1 and 2 for further details). Maps are focusing on south-west Europe, but infections in Scandinavia, Lake Baikal region and USA are also known (Additional files 2 and 4). Map created by authors using Qgis 2.18.4 (QGIS Development Team 2009)

The four species of *Dictyocoela* infecting *G. balcanicus* were not evenly represented when considering both haplogroup diversity and prevalence. *Dictyocoela berillonum* was found in seven individuals from four sites, only in the southern part of the *G. balcanicus* distribution (Fig. 4a, Additional files 1 and 2). While half of the 12 haplogroups of *D. berillonum* from the literature were found in two or more host species (Additional file 2, Fig. 3), the two haplogroups identified in the present study (Dberb01 and b02) were specific to *G. balcanicus*.

*Dictyocoela duebenum* was found in 36 individuals from 11 sites (Additional files 1 and 2) all over the range of *G. balcanicus* (Fig. 4a). Three new haplogroups were found, adding to those already known to be associated with other gammarid species, especially with *G. duebeni* [21, 35]. The three haplogroups infecting *G. balcanicus* were not shared with any of other host species (Fig. 3).

**Fig. 5:**
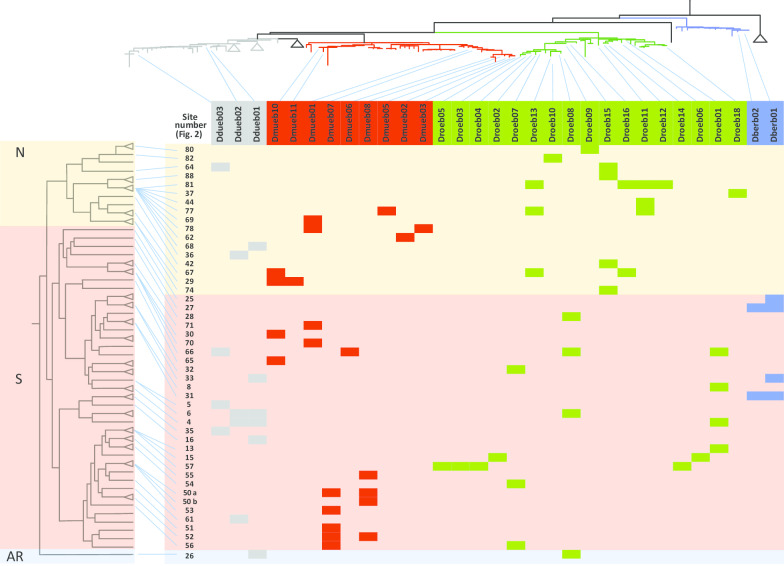
Host-parasite phylogenetic association matrix. Head rows are hosts MOTUs represented by population acronyms and head columns represent parasite haplogroups, with species according to color code, *Dictyocoela duebenum* (grey), *D. muelleri* (red), *D. roeselum* (green) and *D. berillonum* (blue). The background colors of the body of the table represent the three majors host clades (see Fig. 1), *N* North-Eastern (peach), *S* South-Western (pink), and *AR* Ancient Rhodope (blue). Each cell with a color different from the background represent an association, i.e. means that the host in the row is infected by the parasite in the column. Phylogenies of both host and parasite are superimposed, host (left), parasite (top) and links are marked by blue lines. See Fig. 1 and [39] for detailed host phylogeny, and Fig. 3 for parasite phylogeny and haplogroups

*Dictyocoela muelleri* was found in 48 individuals in 17 populations in Romania, Ukraine and Croatia, representing two geographically distinct zones, i.e. the N and S parts of *G. balcanicus* range (Fig. 4b, Additional files 1 and 2). Nine haplogroups were detected in our study, adding to the 29 haplogroups already detected in other gammarid species (Fig. 3, Additional file 2). These nine haplogroups can be grouped in two sets on the phylogenetic tree (Fig. 3). One set included haplogroups clustered within a single clade: Dmueb01, 02, 03, 05, 06, 07 and 08. The geographic distribution of these haplogroups was contrasting, with haplogroups Dmueb06, b07 and b08 restricted to the southern part of the distribution of *G. balcanicus* (Figs. 4b, 5) and haplogroups Dmueb01, b02, b03, b05 present only in the northern part of the host range and associated with the host clade #3 (Figs. 1, 4b, 5). The second set included haplogroups Dmueb10 and b11 found in four Romanian populations (Figs. 3, 4b). They were phylogenetically close to microsporidia infecting *Gammarus roeselii*, but also Ponto-Caspian *Dikerogammarus haemobaphes* and *Pontogammarus robustoides* (Fig. 3). The haplogroups Dmueb01 and b11 were found in hosts’ phylogenetic clades associated with both N and S geographic regions (Figs. 4b, 5).

Finally, *Dictyocoela roeselum* was the most widespread *Dictycoela* in *G. balcanicus*, detected in 46 individuals from 23 sites all over the host range (Figs. 3, 4c, Additional files 1 and 2). *Dictyocoela roeselum* was genetically diverse, accounting for 17 newly identified haplogroups. In addition to these new data, three other haplogroups were already reported for *G. balcanicus* by [24]: MG773218, MG773221 and MG773220, and 17 other haplogroups were already known to infect other gammarid species. Again, each of these new haplogroups was specific to *G. balcanicus*, with the noticeable exception of Droeb18, identical to a haplogroup already found in *G. roeselii* [27] (Additional file 4, Fig. 3). Apart from this exception, others *D. roeselum* haplogroups were clustered into clades infecting only *G. balcanicus* (Fig. 3).

### Co-diversification between *Dictyocoela* and *G. balcanicus*?

**Fig. 6:**
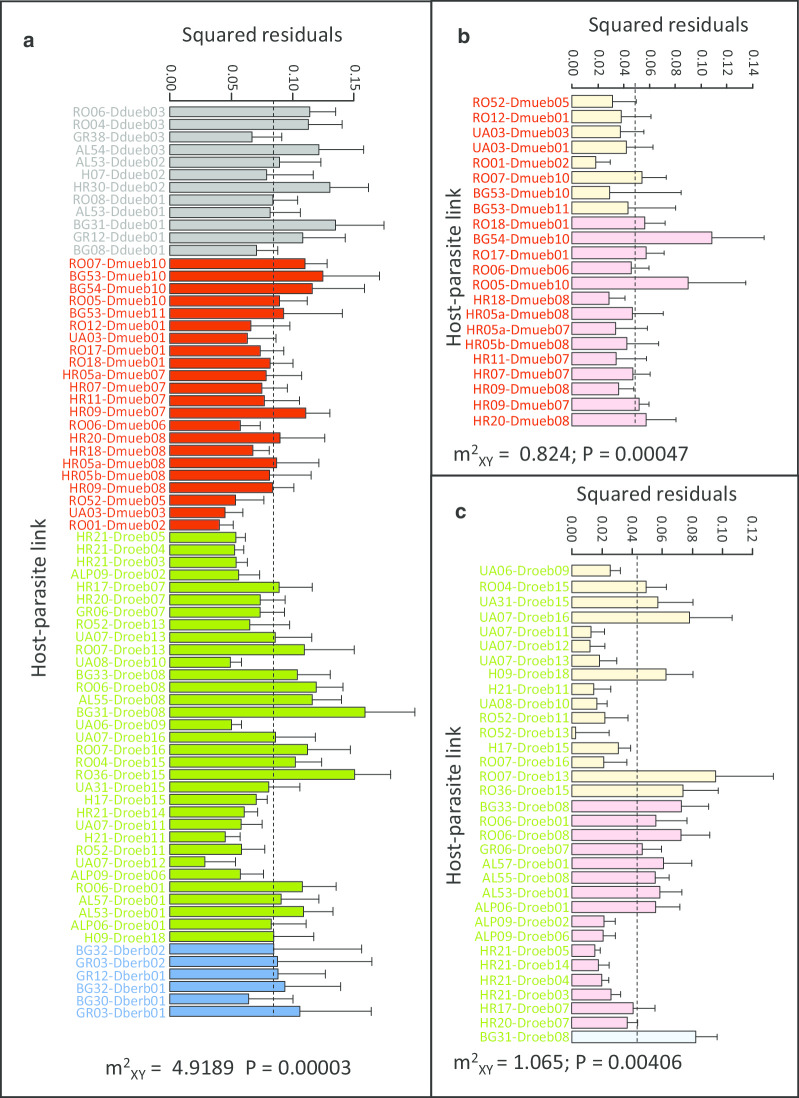
Square residuals for each host-parasite link, contributing to the PACo goodness-of-fit test (m^2^xy) for co-phylogenies between **a** All *Dictyocoela* spp. found in *Gammarus balcanicus*, **b** *D. muelleri* and *G. balcanicus*, **c** *D. roeselum* and *G. balcanicus*. In **a**, colours of the bars are indicating *Dictyocoela* genera (bars were ranked according to the order of appearance in the microsporidia phylogenetic tree, Fig. 3). In **b** and **c**, bars were ranked according to geography, where colours indicates if parasites occurred in the Northern part of the host geographic distribution (peach), in the Southern part (pink) or in the AR part (blue)(see Figs. 1 and 5). The goodness-of-fit (residual sum of squares, m^2^_XY_) and the associated P value are provided. The dotted line shows the median residual value (for information) and error bars show the upper 95% confidence intervals

The PACo analysis revealed that the association between evolutionary divergences of *G. balcanicus* and *Dictyocoela* spp. was not random (P = 0.00003, see Fig. 6a). The individual links with low values of squared residuals, i.e. those contributing the most to the co-phylogeny, were found mostly in *D. muelleri* and *D. roeselum* (see below) (Fig. 6a). However, the squared residuals in links involving the site #26, i.e. the only relict population of the AR host clade (Fig. 1), were high, meaning that they did not contributed to the overall congruence of phylogenies. Similarly, most of the individual links involving *D. duebenum* contributed little to the overall congruence (Fig. 6a).

At the specific level in *Dictyocoela* (at least the species where haplogroups were numerous enough to run an analysis, i.e. *D. muelleri* and *D. roeselum*), associations between host and parasite phylogenies were not random either. It was the case for *D. muelleri* (P = 0.00047, Fig. 6b). Indeed, two groups of host sites shared closely-related *D. muelleri* haplogroups. The first group consisted of localities #62, 69, 77, and 78 located in the N part of the host distribution and phylogeny, the second group consisted of sites #50, 51, 52, 53 and 55, located in the S part of the host distribution/phylogeny (Figs. 2, 5). These two groups contributed importantly to the observed high probability of co-phylogeny, as revealed by their relatively low values of squared residuals (Fig. 6b). Similarly, a strong signal of co-phylogeny was also found between *D. roeselum* and *G. balcanicus* (P = 0.00406, Fig. 6c). The cluster of haplogroups Droeb11 - b12 - b13 was infecting hosts from sites in the northern part of the *G. balcanicus* range, mostly the host phylogenetic clade #3 (Fig. 5). These host-parasite couples generally contributed strongly to the general phylogenetic link (low square residual values, Fig. 6c). A similar high contribution to the host-parasite links was found for the parasite cluster grouping haplogroups Droeb02 b03, b04, b05, b06, b07 and b14 that infected hosts from the southern part of the geographical distribution (Figs. 4c, 5, 6c). Haplogroups Droeb09 and b10 were found only in the Crimean Peninsula (Fig. 4c). They were associated only with the host clade #1 (Figs. 1, 5) and also contributed highly to the host-parasite link (Fig. 6c). Conversely, the haplogroups Droeb01 and b08, while restricted to the southern parts of *G. balcanicus* range, were present in distantly-related host phylogenetic clades and contributed relatively little to the general host-parasite link (Figs. 5, 6c). Droeb08 also infected hosts from the population 26, which, as noted for the analysis involving all *Dictyocoela* species, therefore contributed little to the overall congruence (Fig. 6c). Similarly, Droeb18, associated with only one individual found in Hungary (Fig. 4c), showed a relatively high value of the squared residual (Fig. 6c). This haplogroup was 100% identical to *Dictyocoela* infecting *G. roeselii* (Genbank: AY584252, [24, 31]). It is worth noting that *G. roeselii* is sympatric with *G. balcanicus* in site #37, where this infected individual was found [27].

## Discussion

The exploration of *Dictyocoela* infections in *Gammarus balcanicus* adds 31 haplogroups to this genus of microsporidia. The phylogenetic reconstruction and inclusion to the general phylogeny built by [24] and [27], to which we added recently-published sequences from the Baikal region [19, 26, 28], allowed us to assign these genotypes unambiguously to four already-identified species: *D. muelleri*, *D. roeselum*, *D. duebenum* and *D. berillonum*. No new *Dictyocoela* highly divergent major clade was found in our samples of *Gammarus balcanicus*. However, our new sequences offered new insights for the analysis of a fascinating Russian-stacking-doll pattern of specificity.

At a global level, our expanded dataset confirmed that no strict specificity could be found between *Dictyocoela* species and their hosts. Most species or major clades of *Dictyocoela* were found infecting several host species, some of them being distantly phylogenetically related within amphipods [59]. Few degrees of specialization can be found. The only example is the *Dictyocoela* clade infecting the Baikal amphipod species *Gmelinoides fasciatus*. Some other Baikal *Dictyocoela* infect several gammarid species in this geographic area. In addition, *Gammarus lacustris*, which is present in Lake Baikal system but has a much wider geographic distribution [61], can be infected by both typical Baikal *Dictyocoela* or other *Dictyocoela* strains from other regions (see Fig. 3, see also [20] for an example). Therefore, some *Dictyocoela* strains can be considered as endemic to Lake Baikal system, rather than host-specific, probably after a process of local selection or genetic drift. Conversely, however, *Dictyocoela* species found elsewhere (*D. muelleri* and *D. duebenum*) can infect amphipods from the Baikal group (Fig. 3), confirming the hypothesis of [26] that *Dictyocoela* are circulating among hosts within this geographic area. All other *Dictyocoela* clades/species infected species belonging to Gammarida, but a single parasite species often infect host species belonging to different clades [59]. In addition, several hosts often share a single genotype of the parasite (as estimated by its SSU rDNA sequence), but this rule suffers a number of exceptions (e.g. in *D. roeselum*). In their co phylogenetic analysis, Park et al. [38] showed that there is reasonable evidence for a global *Dictyocoela*-amphipod codivergence, despite a general lack of specificity. Altogether, these results suggest that infection by *Dictyocoela* in amphipods is very ancient (see also [38]). The common ancestor should be at least older than the divergence between Talitrida and Gammarida, i.e. during early Mesozoic, ca. 180–200 MYA [58]. The major diversification of *Dictyocoela* should have taken place before host diversification because most parasite groups infect diverse host groups [38]. The only exception could be *D. roeselum* that infect only hosts of the *Gammarus* genus. This parasite species could therefore have emerged at the same times as the *Gammarus* groups differentiated from other amphipods, i.e. around 50–60 MYA according to [59].

Going down to the level of a single host species (or more precisely to a group of cryptic species, see [59]; [39]), such as *Gammarus balcanicus* studied here, confirms that it can be infected by several *Dictyocoela* species. However, our PACo analyses revealed that diversification of the parasites was not random with respect to host diversification, both between *Dictyocoela* species and at the level of diversification within each parasite species. The degree of host-parasite co-phylogenetic congruence was high between *Dictyocoela* species and *G. balcanicus* cryptic diversity shown by [39]. As noted by [3], methods testing co phylogenies tend to overestimate real host-parasite co-speciations and any observation of congruence between two phylogenetic patterns should be examined with caution. In the case of *G. balcanicus*, we observed some striking patterns of such congruence, notably within *D. roeselum* and *D. muelleri*. We, therefore, think that co-differentiation between strains within these two *Dictyocoela* species and *G. balcanicus* clades could drive the overall *Dictyocoela*-*G. balcanicus* pattern. Indeed, within the two aforementioned parasite species, the divergence between main clades match well the early divergence of the northern and southern (i.e. N and S) host clades. In *D. roeselum*, two parasite groups seem to have split following this pattern (Figs. 4, 5, 6c) and then diversified within the two host lineages and biogeographical regions. Diversification occurred even within populations, as observed in sites ALP09 in Italy (#15) and HR21 in Croatia (#57) (Fig. 4, Additional file 1). The high degree of endemism and local differentiation of the different of *G. balcanicus* (Mamos et al. 2016) probably allowed some local adaptation and red-queen phenomenon of selection, leading to local diversification in parasites [62]. A similar type of pattern was found for *D. muelleri* haplogroups Dmueb07 and 08, which were found only in the southern geographical region of *G. balcanicus*, linked only with the host clade #7 that differentiated there (Figs. 1, 5). A hypothesis alternative to co-differentiation could be that *Dictyocoela* diversity follows a geographical pattern independently of that of *G. balcanicus*, for example following the pattern of a putative intermediate or reservoir hosts, not detected yet because of sample bias. While we cannot exclude that *Dictyocoela* may infect other aquatic organisms (such a hypothesis is worth examining), it seems improbable to us. The only available survey—to our best knowledge—made on 12 macroinvertebrate host taxa in the same river indicates that, while numerous freshwater invertebrates may share some species-level-taxa microsporidian parasite in the same stream, *Dictyocoela* spp. were not found in taxa other than gammarids [14]. Also, even if relatively scarce, investigations in aquatic organisms other than amphipods or fish led to the discovery of several new microsporidian species, but not *Dictyocoela* (e.g. for the most recent [63–70]), contrasting with the studies made on amphipods of different clades where *Dictyocoela* were easily found (e.g. for the most recent [19, 26–28, 38]. The closest relative of *Dictyocoela* spp. is *Diplokaryon legeri* (Figure S2 in [24]), a microsporidium infecting digenean parasite of marine bivalves [65, 71]. Even if hyperparasitism (i.e. parasitic organism infecting a parasite) would be and efficient pathway for host-shifts [68], such a hypothesis is not supported by the current data, *Unikaryon* being too far from *Dictyocoela* phylogenetically and ecologically.

Nevertheless, we found some obvious incongruences between the host and *Dictyocoela* phylogenies. One example is *Dictyocoela duebenum*, for which all detected haplogroups share host genotypes belonging to very different clades, and where most of the individual host-parasite links contributed poorly to the general host-parasite phylogenetic congruence (Fig. 6). *Dictyocoela duebenum* infect a wide range of hosts, where one single haplogroup may be shared between several host species (Additional file 4; [24]). This parasite could therefore be a generalist, characterized by high rate of host shifts. Another noticeable incongruence is found between the two parasites infecting gammarids from the site #26 (Fig. 6a, c), i.e. the relict AR lineage of the most ancient host vicariance (Fig. 1). These parasites are also found to infect individuals belonging to other host clades, from sites located in the southern geographic range of *G. balcanicus*, where site #26 is also located (Figs. 2, 5). This population may, therefore, have been infected by parasites transferred from neighboring sites rather than by parasites having a common phylogenetic history.

De Vienne et al. [3] showed that convincing examples of co-speciation between hosts and parasites seem to be rather an exception than a rule. They suggested that the methods testing co-phylogenies overestimates the phylogenetic links, and they evidenced that, in most cases, the detected congruence corresponded in fact to host shifts followed by specialization instead of co-speciation events. However, even if host shifts are obvious in our dataset, we do not believe that this explains the general pattern of our *Dictyocoela*—*G. balcanicus* phylogenetic congruence. First, as stated earlier, the divergence between the two main groups of parasites match well the early divergence of the hosts ([39], Figs. 1, 4). Second, the intimate link between microsporidia, intracellular parasites, and their hosts, as well as the vertical mode of transmission found in *D. muelleri* and *D. roeselum* (e.g. [31]), may favor co-speciation. It was already evidenced between hosts and mutualistic, vertically-transmitted, symbionts [3]. Third, a co-vicariance has already been suggested for explaining the congruent phylogenies between New-Zealand *Dictyocoela* spp. and their *Paracalliope* spp. hosts [38]. Our results would therefore confirm the pattern observed in New-Zealand fauna. However, since most microsporidia use mixed transmission strategy [12, 34] and because we also evidenced some host shifts in our data, we believe that the term ‘co-speciation’ is too firm to apply to our case study, and we suggest to use ‘co-diversification’. Patterns of co-diversification with diverse degrees of host shifts has been suggested for other host-microsporidia systems, notably Siberian mosquitoes and their microsporidia [8], and *Gammarus roeselii* and *Nosema granulosis* [27]. Microsporidia may therefore be a parasite clade where finding co-diversification would be frequent. Indeed, while such a pattern has been found in some other host-parasite pairs (e.g. [6, 7]), it remains overall quite rare [3].

## Conclusion

Based on all our data, we propose the following scenario for *Dictyocoela* diversification in *G. balcanicus*. *Dictyocoela* parasites, already diversified at the species level, infected common ancestors of *G. balcanicus*, before 15 MYA, the date around which differentiation of *G. balcanicus* began [39]. Then, host-parasite co-diversification could have potentially occurred for each parasite species, following the diversification of the host (Fig. 1). The most apparent vicariance occurred early during the host diversification, separating the two host clades (N and S) inhabiting the northern and southern regions, profoundly impacting host-parasite association. For *D. roeselum* and *D. muelleri*, the host-parasite co-differentiation continued within each region, sometimes at very local scales, after this first major event. However, a number of subsequent horizontal transfers also occurred in the meantime (e.g. *D. roeselum* haplogroups b01 or b08, *D. muelleri* haplogroup b10). At least one parasite species, *D. duebenum*, did not show a strong pattern of co-diversification, and could have kept the potential to infect all host groups. This is perhaps due to its important capacity for host shifts, as proposed by [24]. Therefore, our results point out that the relationships between microsporidia (particularly *Dictyocoela*) and amphipods (particularly Gammarida) could make a good biological model to investigate host-parasite co-diversification. It would be interesting to investigate in-depth some other target species (or group of closely-related species) of amphipod hosts, to understand the relative rates of co diversification *vs.* host shifts. Pan-European species like *Gammarus pulex* or *G. fossarum* could be excellent models as they are geographically widespread [72, 73] and already known to be infected by microsporidia in some parts of their range [20, 26].

## Supplementary information

**Additional file 1:** Details of the *Dictyocoela* spp. infections in the 88 populations investigated over the geographic range of *Gammarus balcanicus.*

**Additional file 2:** Individual-wise data for *Dictyocoela* spp. infections from this study and found in Genbank (NCBI), mainly for fresh and brackish waters Amphipoda species present in Europe and Lake Baikal.

**Additional file 3:** Worksheet 1, Matrices of genetic distances used for the PACo analyses illustrated on Fig. 6. Worksheet 2, Details of the all the PACo analyses.

**Additional file 4:** Maximum-Likelihood phylogenetic reconstruction based on small ribosomal subunit (SSU) rDNA for the microsporidian genus *Dictyocoela* (detailed version of Fig. 3). Maximum-Likelihood phylogenetic reconstruction based on small ribosomal subunit rDNA for the microsporidian genus *Dictyocoela* infecting amphipods. Four taxa including infections in *G. balcanicus* were ascribed a color i.e.* D. duebenum* (grey), *D. muelleri* (red), *D. roeselum* (green) and *D. berillonum sl* (blue). While the three later clades strictly reflect recent reassessment of the genus taxonomy by Bacela-Spychalska et al. (2018), the former one putatively extends this taxon i.e. *D. duebenum sl* (see text and Additional files 2, 5). Sequences used for species formal description by Bacela-Spychalska et al. [24] are indicated with a star (*). Haplogroups from the present study are in bold. *Dictyocoela* sequences from Genbank include the accession number and the host species abbreviated name(s) (see Additional file 2). Values at nodes are bootstrap values > 50%.

**Additional file 5:** Maximum-Likelihood phylogenetic reconstruction based on small ribosomal subunit rDNA, as well as ITS and LSU when available, for taxon sub-sampling of Additional file 4, aiming at providing evidence of support of some key clades. Four taxa including infections in *Gammarus balcanicus* were ascribed a color i.e.* Dictyocoela duebenum* (grey), *D. muelleri* (red), *D. roeselum* (green) and *D. berillonum sl* (blue). Values at nodes are bootstrap values. Specimens representative of diversity and divergence within each clade were used. Sequences used for species formal description by Bacela-Spychalska et al. [24] are indicated with a star (*).

## Data Availability

Material is available at the Department of Invertebrate Zoology and Hydrobiology, University of Lodz, Poland, upon request. GenBank accession numbers of partial SSU rDNA sequences generated in this study are MT932330-MT932461.

## Abbreviations

BIN: Barcode Index Number
BOLD: Barcode of Life Data system
COI: Cytochrome oxydase I gene
MYA: Million years ago
MOTU: Molecular operational taxonomic unit
SSU rDNA: Small subunit ribosomal gene
PACo: Procrustean Approach to Co-phylogeny
