# Supplementary figures and images for: Dictyocoela microsporidia diversity and co-diversification with their host, a gammarid species complex (Crustacea, Amphipoda) with an old history of divergence and high endemic diversity
Source: BMC Evol Biol. 2020 Nov 11;20:149. doi: 10.1186/s12862-020-01719-z (PMC7659068; doi:10.1186/s12862-020-01719-z)

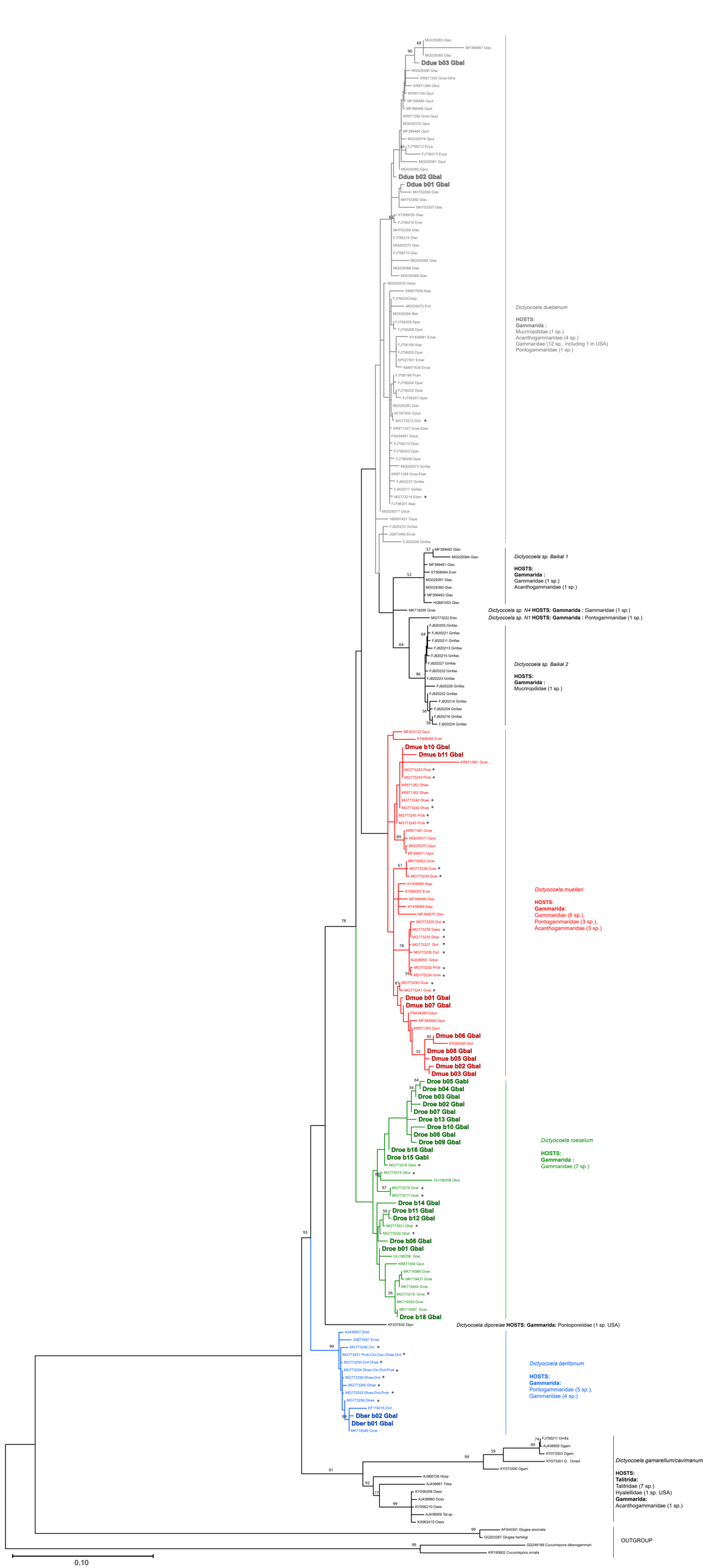

Supplement: Supplementary file 4 — Additional file 4: Maximum-Likelihood phylogenetic reconstruction based on small ribosomal subunit (SSU) rDNA for the microsporidian genus Dictyocoela (detailed version of Fig. 3). Maximum-Likelihood phylogenetic reconstruction based on small ribosomal subunit rDNA for the microsporidian genus Dictyocoela infecting amphipods. Four taxa including infections in G. balcanicus were ascribed a color i.e. D. duebenum (grey), D. muelleri (red), D. roeselum (green) and D. berillonum sl (blue). While the three later clades strictly reflect recent reassessment of the genus taxonomy by Bacela-Spychalska et al. (2018), the former one putatively extends this taxon i.e. D. duebenum sl (see text and Additional files 2, 5). Sequences used for species formal description by Bacela-Spychalska et al. [24] are indicated with a star (*). Haplogroups from the present study are in bold. Dictyocoela sequences from Genbank include the accession number and the host species abbreviated name(s) (see Additional file 2). Values at nodes are bootstrap values > 50%. [file 12862_2020_1719_MOESM4_ESM.pdf]

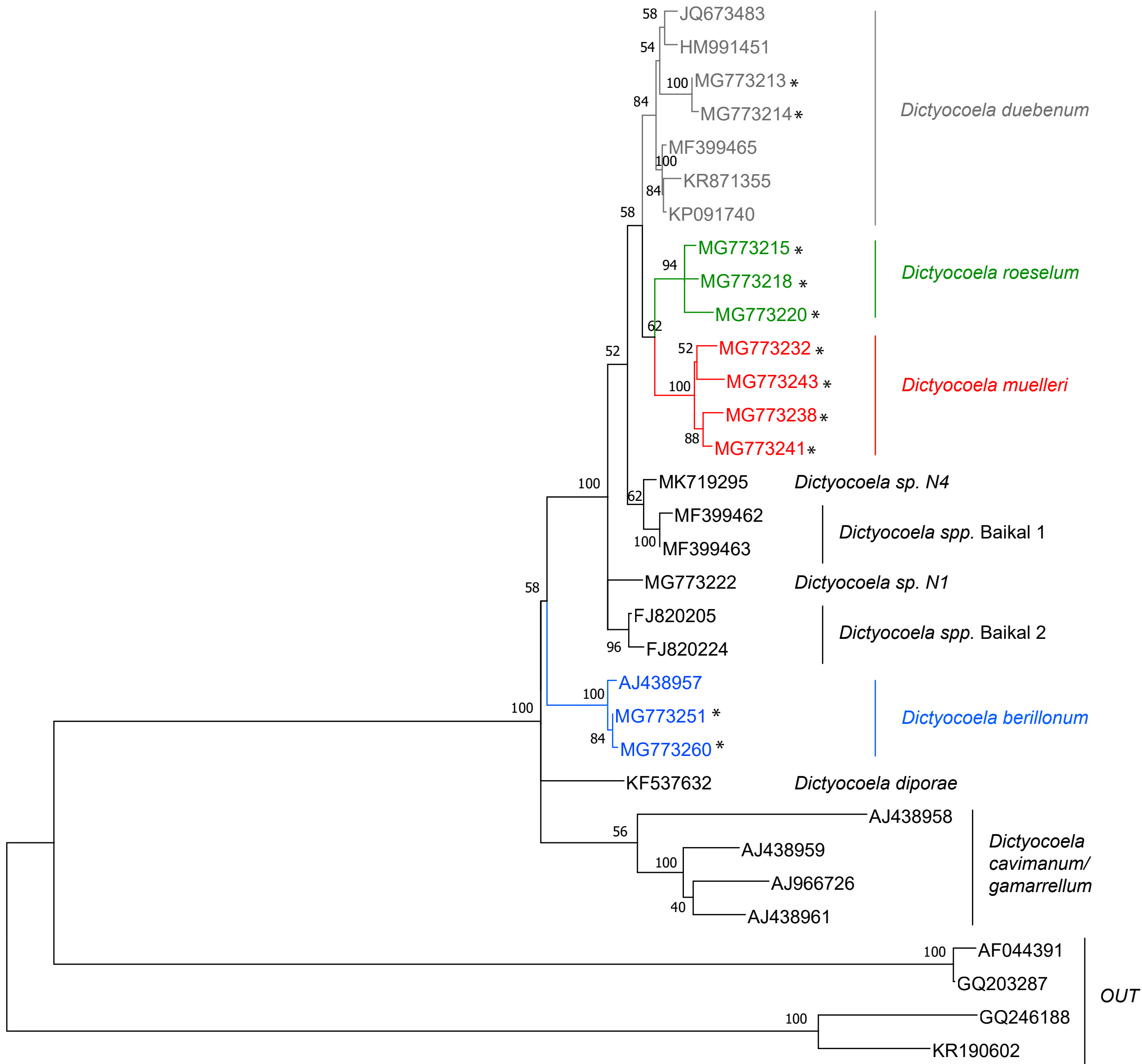

Supplement: Supplementary file 5 — Additional file 5: Maximum-Likelihood phylogenetic reconstruction based on small ribosomal subunit rDNA, as well as ITS and LSU when available, for taxon sub-sampling of Additional file 4, aiming at providing evidence of support of some key clades. Four taxa including infections in Gammarus balcanicus were ascribed a color i.e. Dictyocoela duebenum (grey), D. muelleri (red), D. roeselum (green) and D. berillonum sl (blue). Values at nodes are bootstrap values. Specimens representative of diversity and divergence within each clade were used. Sequences used for species formal description by Bacela-Spychalska et al. [24] are indicated with a star (*). [file 12862_2020_1719_MOESM5_ESM.pdf]
